# Supplementary material for: Growth of lithium-indium dendrites in all-solid-state lithium-based batteries with sulfide electrolytes
Source: Nat Commun. 2021 Nov 29;12:6968. doi: 10.1038/s41467-021-27311-7 (PMC8630065; doi:10.1038/s41467-021-27311-7)
Supplement: Supplementary file 1 — Supplementary Information [file 41467_2021_27311_MOESM1_ESM.pdf]

Supplementary Information

Growth of Lithium-Indium Dendrites in All-Solid-State Lithium-based Batteries with Sulfide Electrolytes

Shuting Luo[1‡], Zhenyu Wang[2‡], Xuelei Li[3], Xinyu Liu[2], Haidong Wang[1], Weigang Ma[1], Lianqi Zhang[3], Lingyun Zhu[2]*, Xing Zhang[1]*

[1] Key Laboratory for Thermal Science and Power Engineering of Ministry of Education, Department of Engineering Mechanics, Tsinghua University, Beijing 100084, China

[2] Guilin Electrical Equipment Scientific Research Institute Co. Ltd., Guilin 541004, Guangxi, China

[3] School of Materials Science and Engineering, Tianjin University of Technology, Tianjin 300384, China.

[‡]These authors contributed equally to this work: Shuting Luo, Zhenyu Wang

*These authors jointly supervised this work: Lingyun Zhu, Xing Zhang

*Correspondence: zhuly@glesi.com.cn, x-zhang@tsinghua.edu.cn

Inventory of supplementary information

S1 XRD patterns of the synthesized Li_6PS_5Cl (LPSCl) and standard LPSCl.

S2 SEM image and EDX mapping of P, S, Cl for electrolyte LPSCl.

S3 Nyquist plot of the synthesized LPSCl solid electrolyte at 25 ℃.

S4 STEM image and EDX mapping of a thin sheet of LNO@NCM622.

S5 Cross-sectional SEM image of the prepared Li-In alloy anode.

S6 Nine sets of repeated experiments for the cells Li-In|LPSCl|LNO@NCM622.

S7 SEM image and EDX mapping of cathode-electrolyte interface for the cell with 897 cycles.

S8 Long-term cycling performance of the liquid cell Li-In|$LiPF_6$|NCM622.

S9 SEM images of Li-In anode in the cycled liquid cell.

S10 Long-term cycling performance of the cells with LGPS and LPS electrolyte.

S11 SEM images of electrolyte-anode interface for the cycled cells with LGPS and LPS electrolyte.

S12 Evolutions of the radial distribution functions of Li-P, Li-S and Li-Cl at LPSCl-In interface.

S13 Evolutions of EIS and corresponding internal resistance of the cell In|LPSCl|In for one week.

S14 Long-term cycling performance of Li-In|LPSCl|LNO@NCM622 at 2 mA cm^{-2}.

S15 Cross-sectional SEM images of the cells at 2 mA cm^{-2} and 3.8 mA cm^{-2}, respectively.

S16 Ragone plots for the cells employing sulfide electrolytes cycled at closed room temperature.

S17 Cycling performance of the cell Li|LPSCl|LNO@NCM622.

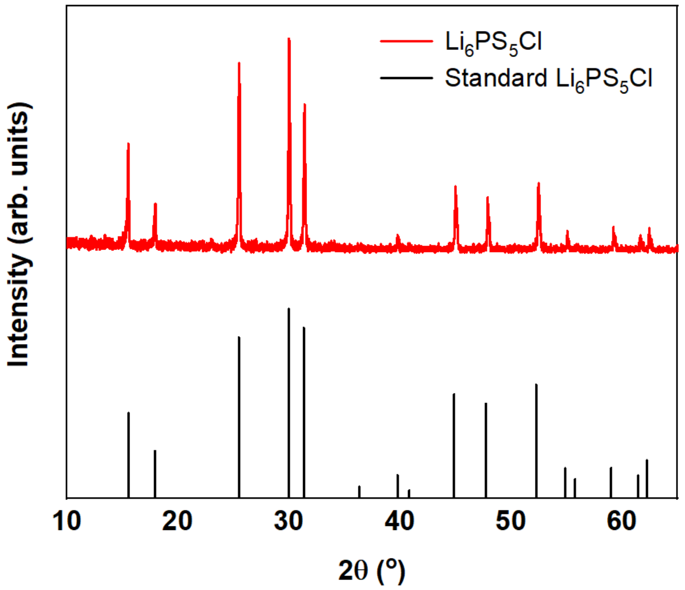

Supplementary Figure 1 XRD patterns of the synthesized Li_6PS_5Cl (LPSCl) and standard LPSCl.

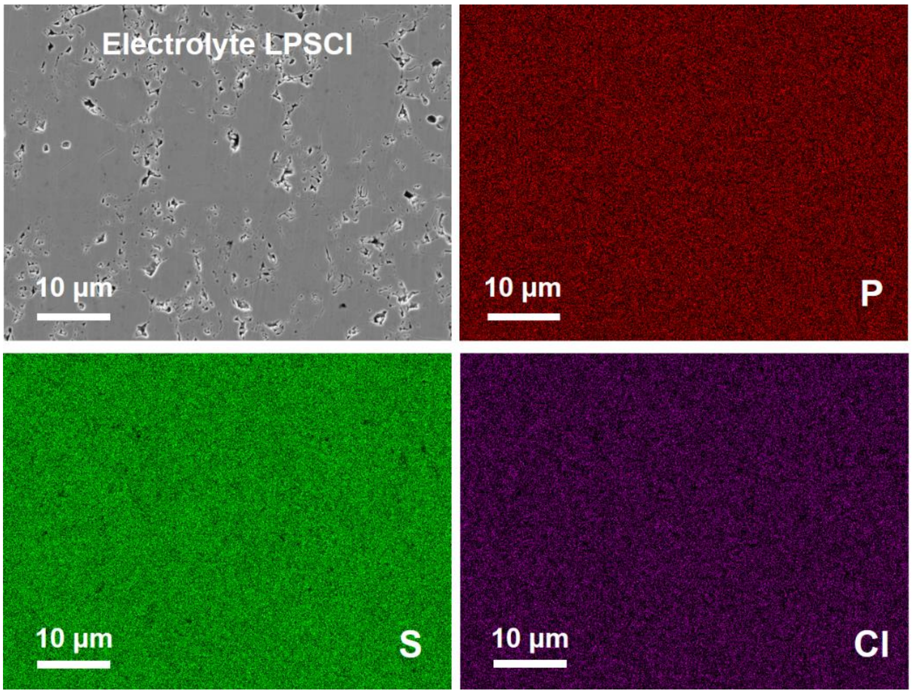

Supplementary Figure 2 SEM image and EDX mapping of P, S, Cl for electrolyte LPSCl.

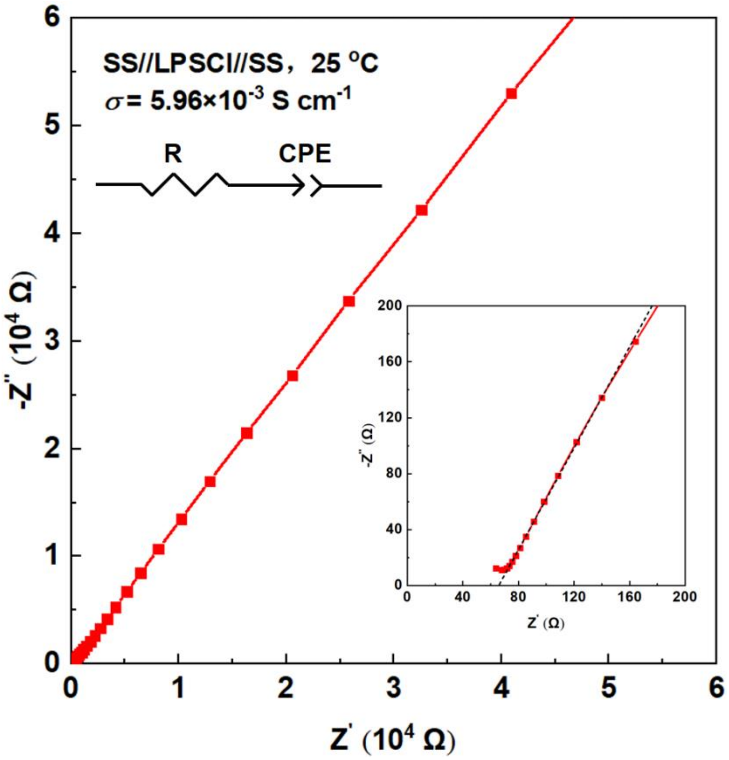

Supplementary Figure 3 Nyquist plot of the synthesized LPSCl solid electrolyte at 25 ℃.

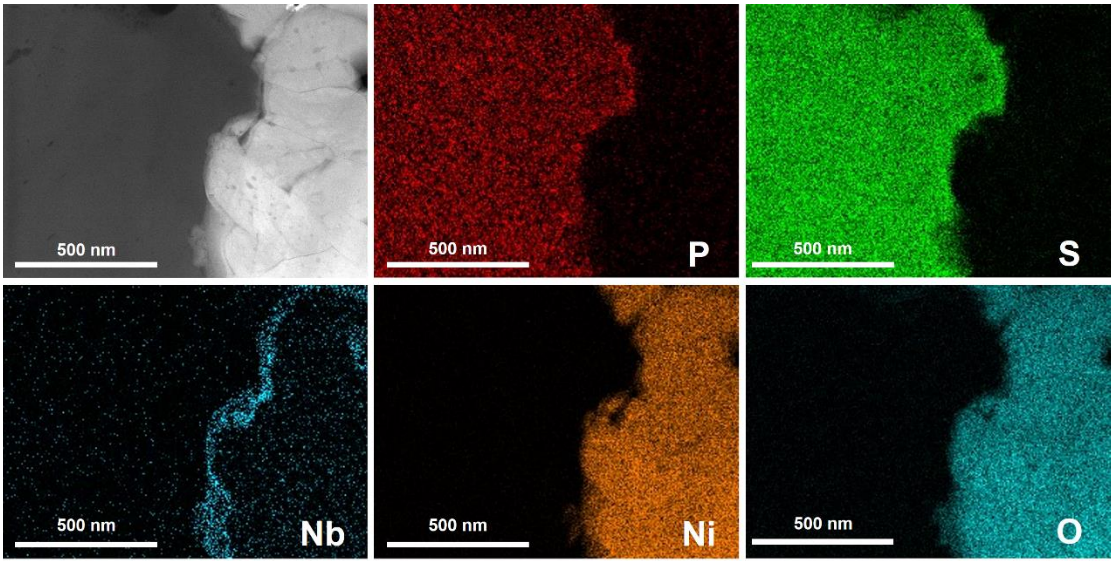

Supplementary Figure 4 STEM image and EDX mapping of a thin sheet of LNO@NCM622.

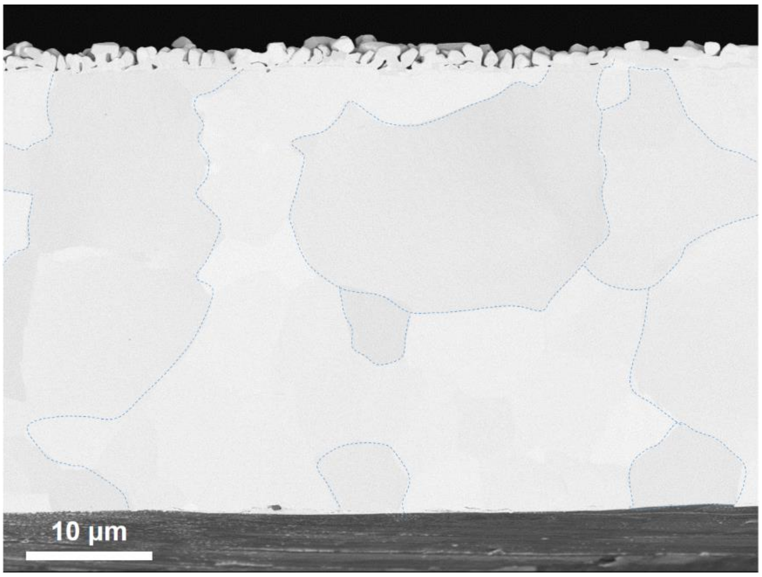

Supplementary Figure 5 Cross-sectional SEM image of the prepared Li-In alloy anode.

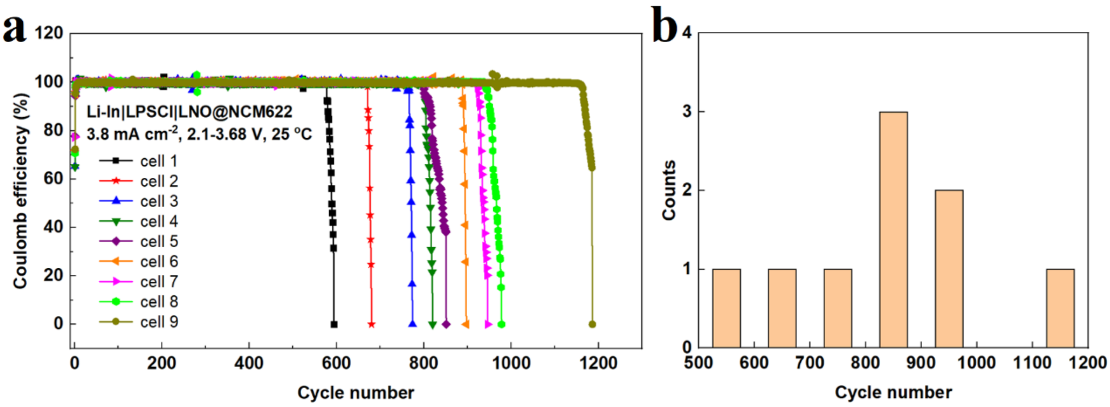

Supplementary Figure 6 Nine sets of repeated experiments for the cells Li-In|LPSCl|LNO@NCM622. **a** Long cycling performance at 3.8 mA cm^{-2} at 25 °C. **b** Distribution of cycling life for the nine cells.

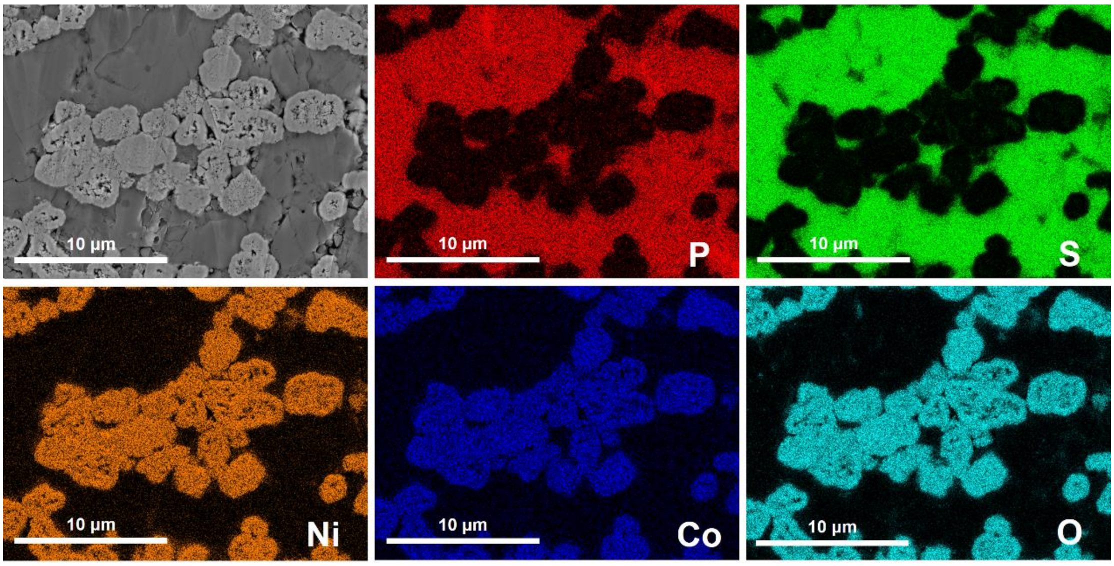

Supplementary Figure 7 SEM image and EDX mapping of P, S, Ni, Co and Mn for cathode-electrolyte interface for the cell with 897 cycles.

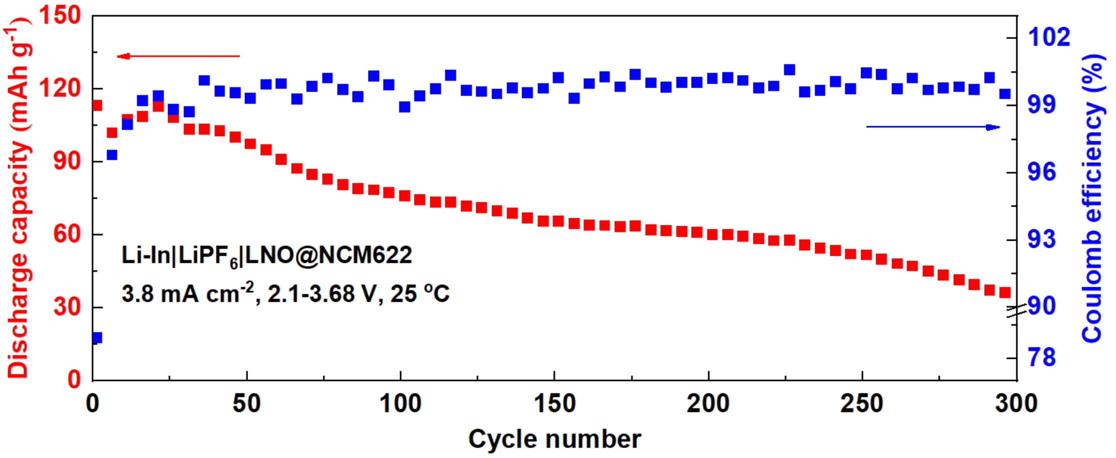

Supplementary Figure 8 Long-term cycling performance of the liquid cell Li-In|LiPF$_6$|NCM622 at 3.8 mA cm^{-2} at 25 °C.

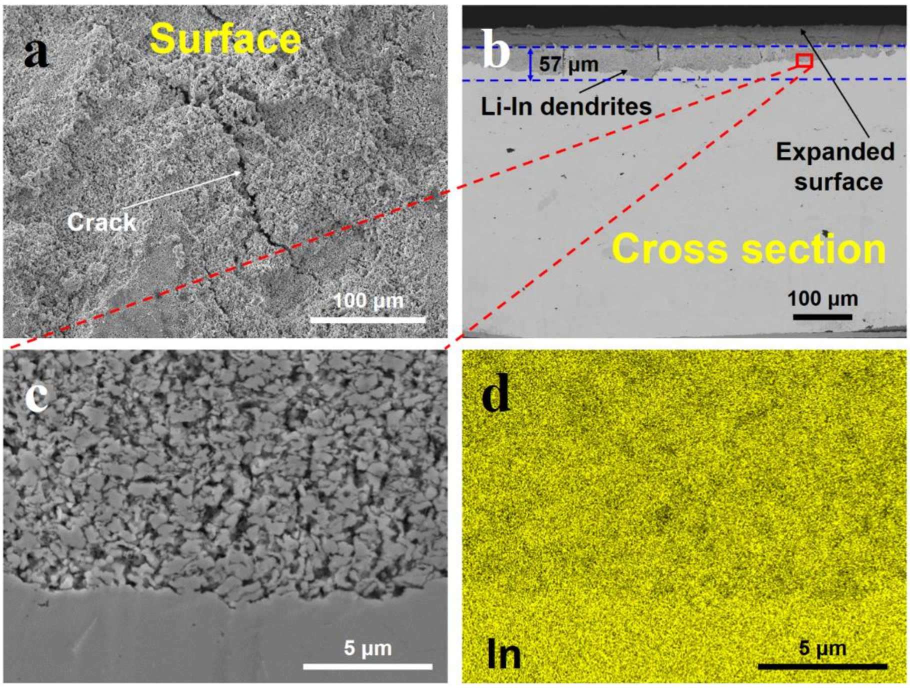

Supplementary Figure 9 SEM images for the Li-In anode cycled 300 times in liquid cell. **a** SEM image for the surface of Li-In anode. **b** SEM image for the cross section of Li-In anode. **c** Magnified SEM image of Li-In dendrites in red boxed area. **d** Corresponding EDX mapping of element In in red boxed area.

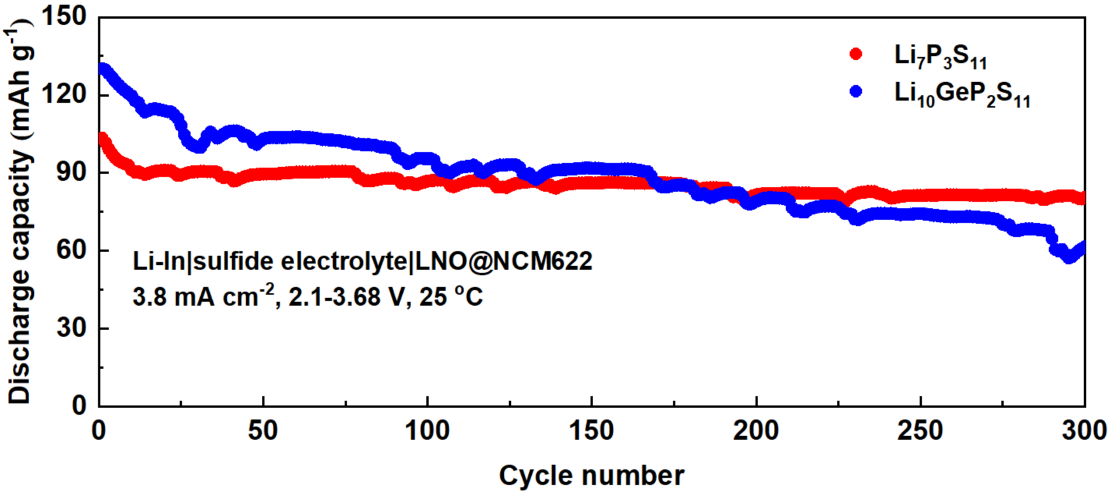

Supplementary Figure 10 Long-term cycling performance of the cells Li-In|LGPS|LNO@NCM622 and Li-In|LPS|LNO@NCM622 at 3.8 mA cm^{-2} at 25 °C.

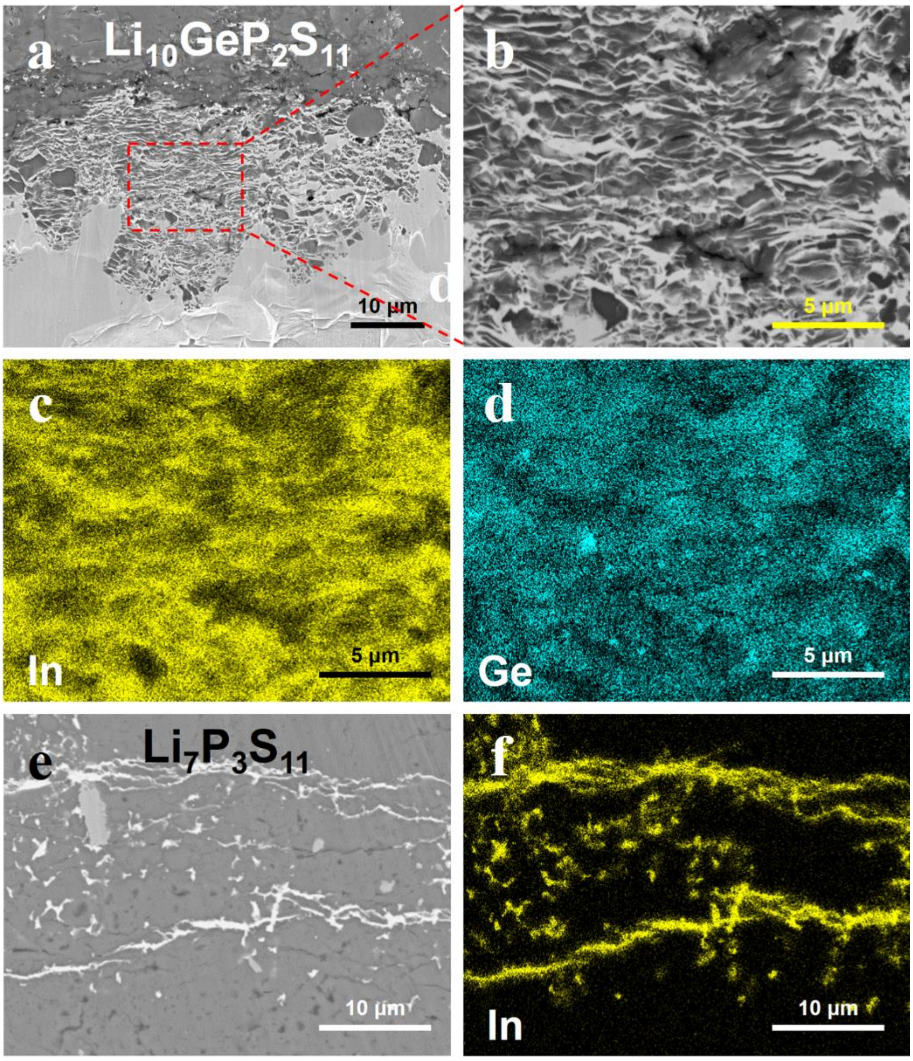

Supplementary Figure 11 Cross-sectional SEM images of the electrolyte-anode interface after 300 cycles. **a** Cross-sectional SEM image and **b** magnified SEM image of Li-In dendrites in LGPS electrolyte. EDX mapping of **c** In and **d** Ge in the red boxed area. **e** SEM image of Li-In dendrites in LPS electrolyte. **f** Corresponding EDX mapping of In.

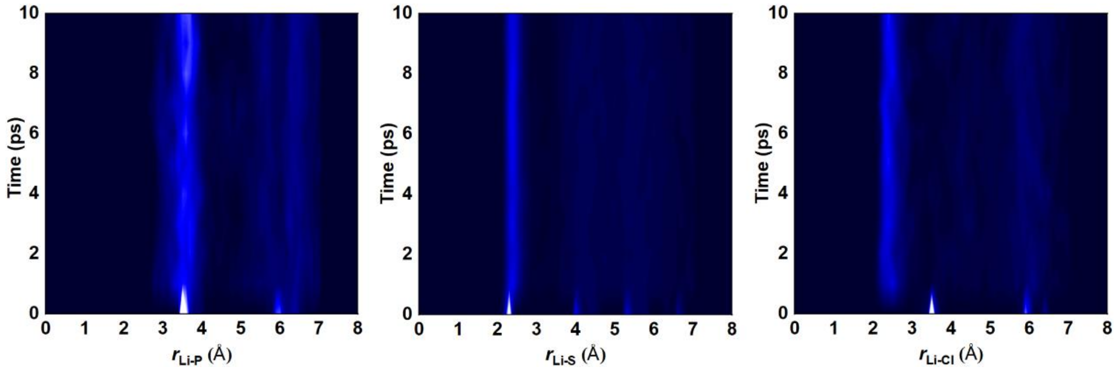

Supplementary Figure 12 Evolutions of the radial distribution functions (RDFs) vs. AIMD simulation time at room temperature (300 K), where RDFs of Li-P, Li-S and Li-Cl are at LPSCl-In interface.

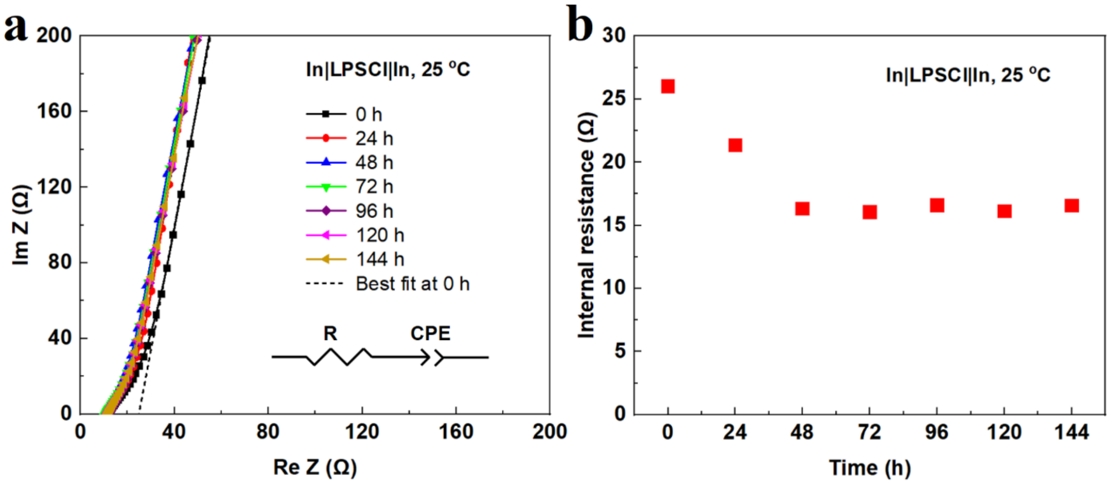

Supplementary Figure 13 Evolutions of electrochemical impedance spectrum and corresponding internal resistance of the cell In|LPSCl|In for one week. The equivalent circuit is shown in inset. The maximum error between the raw and fitted data is 2.6%.

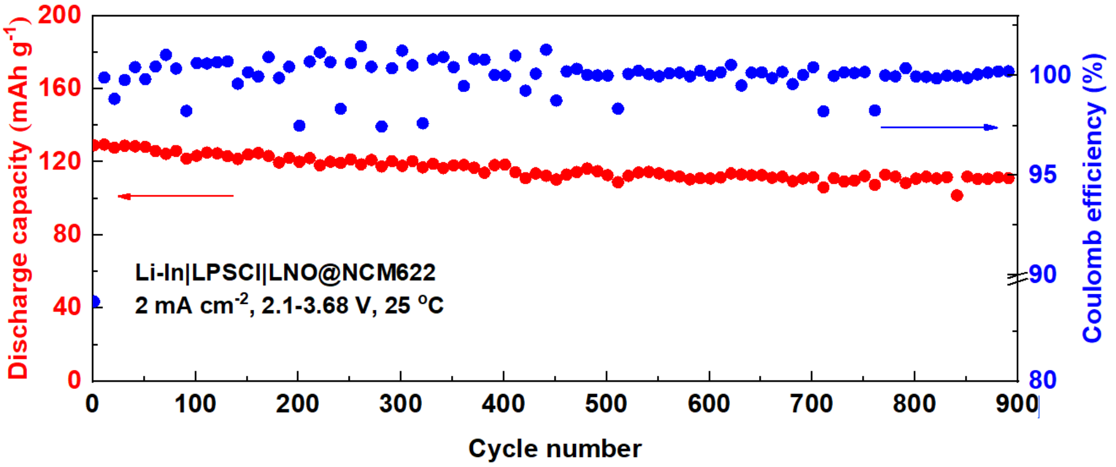

Supplementary Figure 14 Long-term cycling performance of Li-In|LPSCl|LNO@NCM622 at 2 mA cm^{-2} at 25 °C.

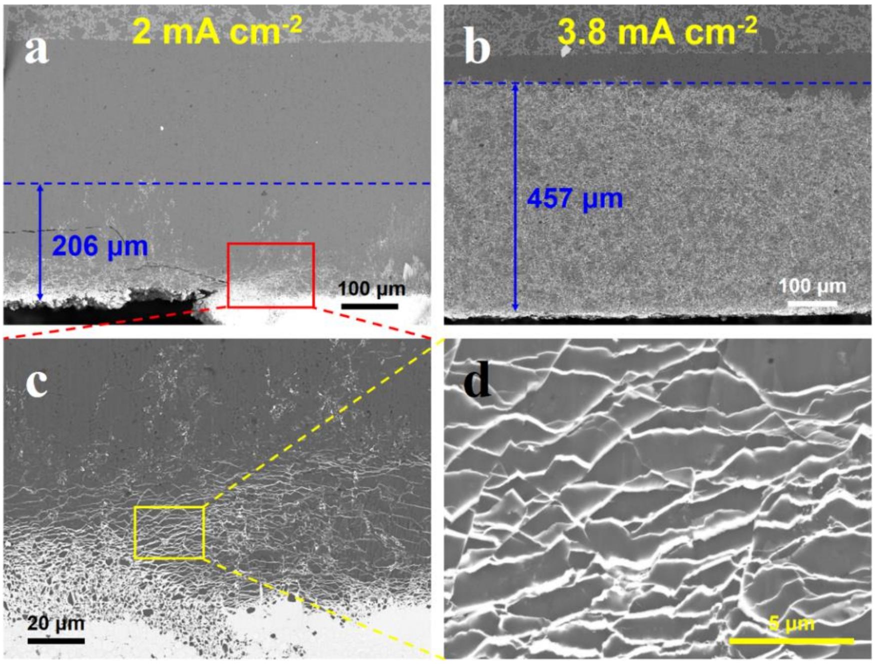

Supplementary Figure 15 Cross-sectional SEM images of the cells **a** at 2 mA cm^{-2} and **b** at 3.8 mA cm^{-2}, respectively. Magnified SEM images of **c** the red boxed area and **d** the yellow boxed area.

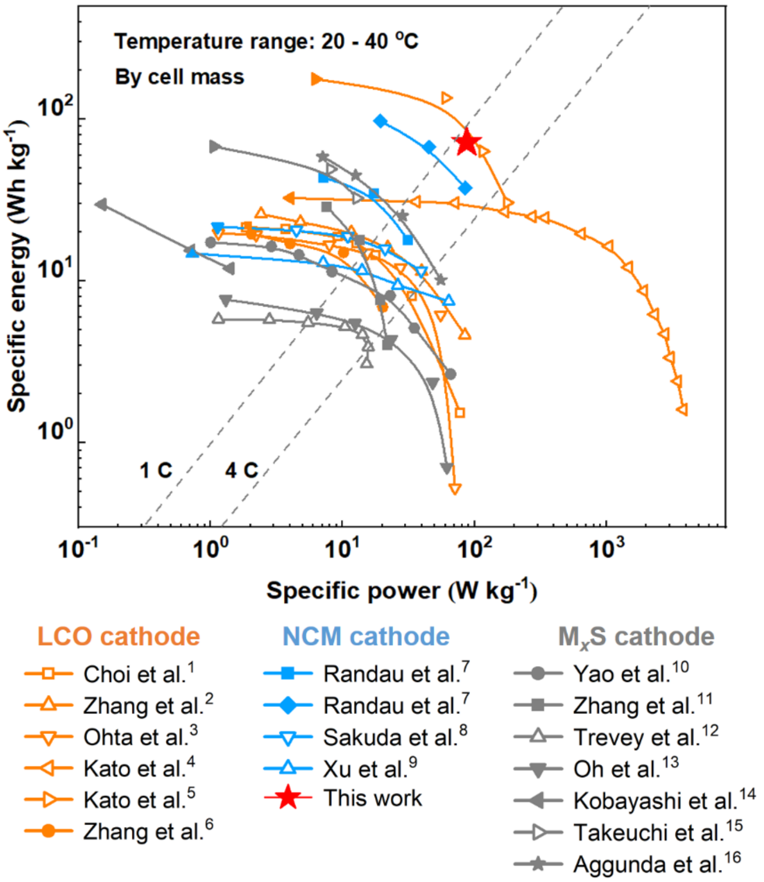

Supplementary Figure 16 Ragone plots for the cells employing sulfide electrolytes cycled at closed room temperature. The specific energy and power were delivered during discharging, normalized by the cell mass. ASSLBs with $LiCoO_2$ (LCO)[1-6], $LiNi_xCo_yM_{1-x-y}$ (NCM)[7-9] and M_xS (M = Co, Ni, Ti, Li, Fe, Cu)[10-16] are compared. Filled symbol indicates that the cell was charged and discharged at equal current. Empty symbol indicates the cell was charged at a certain current and discharged at different rates.

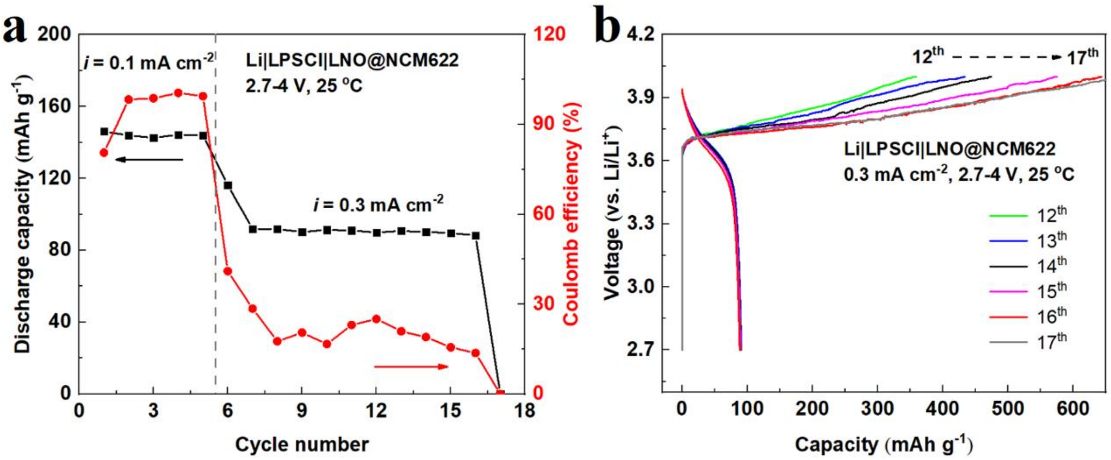

Supplementary Figure 17 Cycling performance of the cell Li|LPSCl|LNO@NCM622. **a** Variation of discharge capacity versus cycle number for the cell at 0.3 mA cm^{-2} at 25 °C. **b** Galvanostatic charge-discharge profiles from the 12th to the 17th cycle.

References

1. Choi, Y. E. et al. Coatable Li_4SnS_4 solid electrolytes prepared from aqueous solutions for all-solid-state lithium-ion batteries. *Chemsuschem* **10**, 2605-2611 (2017).

2. Zhang, W. B. et al. Interfacial processes and influence of composite cathode microstructure controlling the performance of all-solid-state lithium batteries. *ACS Appl. Mater. Inter.* **9**, 17835-17845 (2017).

3. Ohta, N. et al. Enhancement of the high-rate capability of solid-state lithium batteries by nanoscale interfacial modification. *Adv. Mater.* **18**, 2226-2229 (2006).

4. Kato, Y. et al. High-power all-solid-state batteries using sulfide superionic conductors. *Nat. Energy* **1**, (2016).

5. Kato, Y. et al. All-solid-state batteries with thick electrode configurations. *J. Phys. Chem. Lett.* **9**, 607-613 (2018).

6. Zhang, Z. H. et al. Interface re-engineering of $Li_{10}GeP_2S_{12}$ electrolyte and lithium anode for all-solid-state lithium batteries with ultralong cycle life. *ACS Appl. Mater. Inter.* **10**, 2556-2565 (2018).

7. Randau, S. et al. Benchmarking the performance of all-solid-state lithium batteries. *Nat. Energy* **5**, 259-270 (2020).

8. Sakuda, A. et al. Electrode morphology in all-solid-state lithium secondary batteries consisting of $LiNi_{1/3}Co_{1/3}Mn_{1/3}O_2$ and Li_2S-P_2S_5 solid electrolytes. *Solid State Ionics* **285**, 112-117 (2016).

9. Xu, X. X. et al. Self-organized core-shell structure for high-power electrode in solid-state lithium batteries. *Chem. Mater.* **23**, 3798-3804 (2011).

10. Yao, X.Y. et al. High-energy all-solid-state lithium batteries with ultralong cycle life. *Nano Lett.* **16**, 7148-7154 (2016).

11. Zhang, Q. et al. Nickel sulfide anchored carbon nanotubes for all-solid-state lithium batteries with enhanced rate capability and cycling stability. *J. Mater. Chem. A* **6**, 12098-12105 (2018).

12. Trevey, J. E. et al. High power nanocomposite TiS_2 cathodes for all-solid-state lithium batteries. *J. Electrochem. Soc.* **158**, A1282-A1289 (2011).

13. Oh, D.Y. et al. All-solid-state lithium-ion batteries with TiS_2 nanosheets and sulphide solid

electrolytes. *J. Mater. Chem. A* **4**, 10329-10335 (2016).
14. Kobayashi, T. et al. All solid-state battery with sulfur electrode and thio-LISICON electrolyte. *J. Power Sources* **182**, 621-625 (2008).
15. Takeuchi, T. et al. Application of graphite-solid electrolyte composite anode in all-solid-state lithium secondary battery with Li_2S positive electrode. *Solid State Ionics* **262**, 138-142 (2014).
16. Santhosha, A. L. et al. Macroscopic displacement reaction of copper sulfide in lithium solid-state batteries. *Adv. Energy Mater.* **10**, (2020).
