# Supplementary material for: Growth of lithium-indium dendrites in all-solid-state lithium-based batteries with sulfide electrolytes
Source: Nat Commun. 2021 Nov 29;12:6968. doi: 10.1038/s41467-021-27311-7 (PMC8630065; doi:10.1038/s41467-021-27311-7)
Supplement: Supplementary file 3 — Reporting Summary [file 41467_2021_27311_MOESM3_ESM.pdf]

nature portfolio

Corresponding author(s): Xing Zhang

Last updated by author(s): Oct 30, 2021

Reporting Summary

Nature Portfolio wishes to improve the reproducibility of the work that we publish. This form provides structure for consistency and transparency in reporting. For further information on Nature Portfolio policies, see our Editorial Policies and the Editorial Policy Checklist.

Statistics

For all statistical analyses, confirm that the following items are present in the figure legend, table legend, main text, or Methods section.

n/a	Confirmed	
☐	☒	The exact sample size (*n*) for each experimental group/condition, given as a discrete number and unit of measurement
☐	☒	A statement on whether measurements were taken from distinct samples or whether the same sample was measured repeatedly
☐	☒	The statistical test(s) used AND whether they are one- or two-sided<br>*Only common tests should be described solely by name; describe more complex techniques in the Methods section.*
☐	☒	A description of all covariates tested
☐	☒	A description of any assumptions or corrections, such as tests of normality and adjustment for multiple comparisons
☐	☒	A full description of the statistical parameters including central tendency (e.g. means) or other basic estimates (e.g. regression coefficient) AND variation (e.g. standard deviation) or associated estimates of uncertainty (e.g. confidence intervals)
☒	☐	For null hypothesis testing, the test statistic (e.g. *F*, *t*, *r*) with confidence intervals, effect sizes, degrees of freedom and *P* value noted<br>*Give P values as exact values whenever suitable.*
☒	☐	For Bayesian analysis, information on the choice of priors and Markov chain Monte Carlo settings
☒	☐	For hierarchical and complex designs, identification of the appropriate level for tests and full reporting of outcomes
☒	☐	Estimates of effect sizes (e.g. Cohen's *d*, Pearson's *r*), indicating how they were calculated

Our web collection on statistics for biologists contains articles on many of the points above.

Software and code

Policy information about availability of computer code

Data collection	The data related to AIMD were calculated and collected using vasp.5.3.2.
Data analysis	The data was analyzed using VMD1.9.3, vesta-win64, origin 2018, excel 2013 and PowerPoint 2013.

For manuscripts utilizing custom algorithms or software that are central to the research but not yet described in published literature, software must be made available to editors and reviewers. We strongly encourage code deposition in a community repository (e.g. GitHub). See the Nature Portfolio guidelines for submitting code & software for further information.

Data

Policy information about availability of data

All manuscripts must include a data availability statement. This statement should provide the following information, where applicable:

- Accession codes, unique identifiers, or web links for publicly available datasets
- A description of any restrictions on data availability
- For clinical datasets or third party data, please ensure that the statement adheres to our policy

All the data that support the findings of this study are freely available in the Figshare repository (DOI: 10.6084/m9.figshare.15021981)

Field-specific reporting

Ecological, evolutionary & environmental sciences study design

All studies must disclose on these points even when the disclosure is negative.

Study description	In this work, vigorous growth of Li-In dendrites in SSE is discovered when a full cell ($LiNi_{0.6}Co_{0.2}Mn_{0.2}O_2$//$Li_6PS_5Cl$//Li-In) is cycled in high current and high cathode loading, which will induce short circuit after a long cycling. The morphology and growth mechanism of Li-In dendrites are revealed by experiments and calculations. The differences between Li and Li-In dendrites are systematically compared.
Research sample	$LiNbO_3$ coated $LiNi_{0.6}Co_{0.2}Mn_{0.2}O_2$, sulfide electrolyte ($Li_6PS_5Cl$, $Li_{10}GeP_2S_{11}$ and $Li_7P_3S_{11}$) and Li-In alloy were used as cathode, electrolyte and anode, respectively. All the electrochemical measurements were performed in glove box without any harm to the environment and human body.
Sampling strategy	Nine sets of repeated experiments with the same testing conditions were performed for the cell $LiNi_{0.6}Co_{0.2}Mn_{0.2}O_2$//$Li_6PS_5Cl$//Li-In to prove the universality of dendrite growth.
Data collection	The electrochemical data were collected using electrochemical workstation (EIS, Bio-Logic VSP-300) and LAND CT3001A. The sample cross section was polished using a cross section polisher (JEOL, IB-19520CCP, Japan). Scanning electron microscope (SEM) equipped with energy-dispersive X-ray spectroscopy (EDX) (JEOL, JSM-7900F, Japan) was used to analyze the cross-sectional morphology of the cell. The microstructure of the tested sample was characterized by cryo-scanning transmission electron microscope (cryo-STEM, HD2700, Hitachi) at -100 °C. The data related to interface reaction were calculated and collected using vasp.5.3.2.℃
Timing and spatial scale	The cycling data of the full cell was collected from February 10 to May 26, 2020. The cell was charged to 3.68V at a constant current mode (3.8 mA cm^{-2}), then charged at constant voltage mode for 15 minutes and finally discharged at 3.8 mA cm^{-2} to 2.1 V. The data were recorded every minute. The diameter and thickness of the cell is 10 mm and 500 µm, respectively.
Data exclusions	No data were excluded from the analyses.
Reproducibility	Nine sets of repeated experiments with the same testing conditions were performed for the cell $LiNi_{0.6}Co_{0.2}Mn_{0.2}O_2$//$Li_6PS_5Cl$//Li-In for further verification. All the cells have a short circuit after a long cycling with cell life mainly distributed in the range of 800~1000 cycles, which demonstrates the universality of cell failure in ASSLBs with Li-In anode. Similarly, Li-In dendrites were also observed in liquid cell and solid cells with other typical sulfide electrolytes $Li_{10}GeP_2S_{11}$ and $Li_7P_3S_{11}$.
Randomization	This is not relevant to our study because our experiments does not involve grouping.
Blinding	Blinding was not relevant to our study because our experiments does not involve grouping.

Did the study involve field work? ☐ Yes ☒ No

Reporting for specific materials, systems and methods

We require information from authors about some types of materials, experimental systems and methods used in many studies. Here, indicate whether each material, system or method listed is relevant to your study. If you are not sure if a list item applies to your research, read the appropriate section before selecting a response.

Materials & experimental systems

n/a	Involved in the study	
☒	☐	Antibodies
☒	☐	Eukaryotic cell lines
☒	☐	Palaeontology and archaeology
☒	☐	Animals and other organisms
☒	☐	Human research participants
☒	☐	Clinical data
☒	☐	Dual use research of concern

Methods

n/a	Involved in the study	
☒	☐	ChIP-seq
☒	☐	Flow cytometry
☒	☐	MRI-based neuroimaging
